# Supplementary material for: Rapid ionic current phenotyping (RICP) identifies mechanistic underpinnings of iPSC-CM AP heterogeneity
Source: bioRxiv. 2023 Aug 18:2023.08.16.553521. Preprint. [Version 1] doi: 10.1101/2023.08.16.553521 (PMC10461967; doi:10.1101/2023.08.16.553521)
Supplement: Supplement 1 [file media-1.pdf]

Supplementary Material

Rapid ionic current phenotyping (RICP) identifies mechanistic underpinnings of iPSC-CM AP heterogeneity

Alexander P. Clark[1], Siyu Wei[2], Kristin Fullerton[3],
Trine Krogh-Madsen,[3,4] David J. Christini[1,2,*]

---

1 Supplementary figures

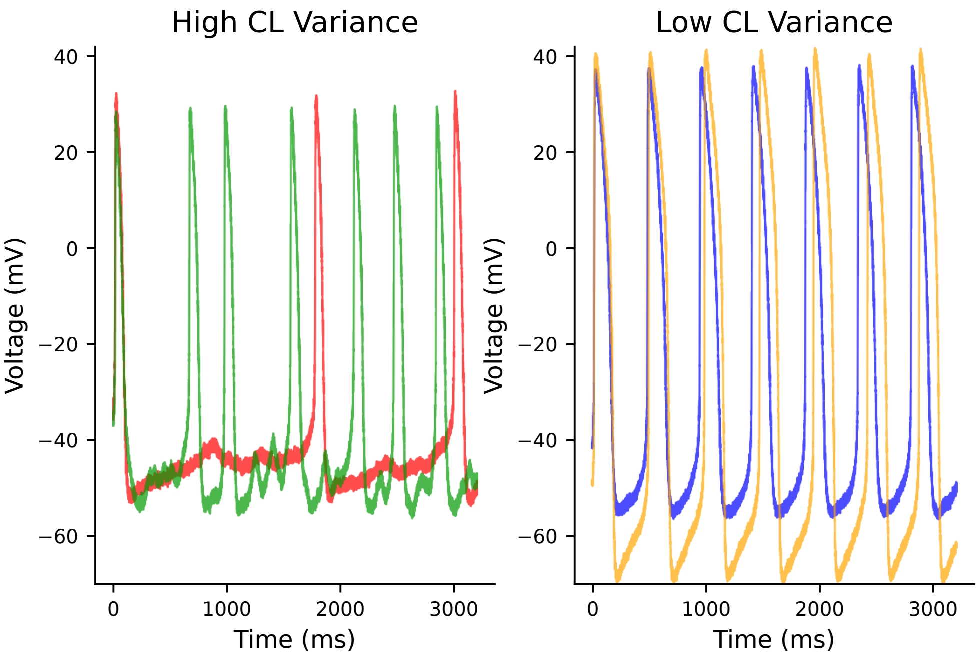

Figure 1: **Cells with high (left) and low (right) variance in cycle length**. High-variance cells have an irregular diastolic depolarization ramp and varying takeoff potential, while low-variance cells show consistent, very regular AP morphologies.

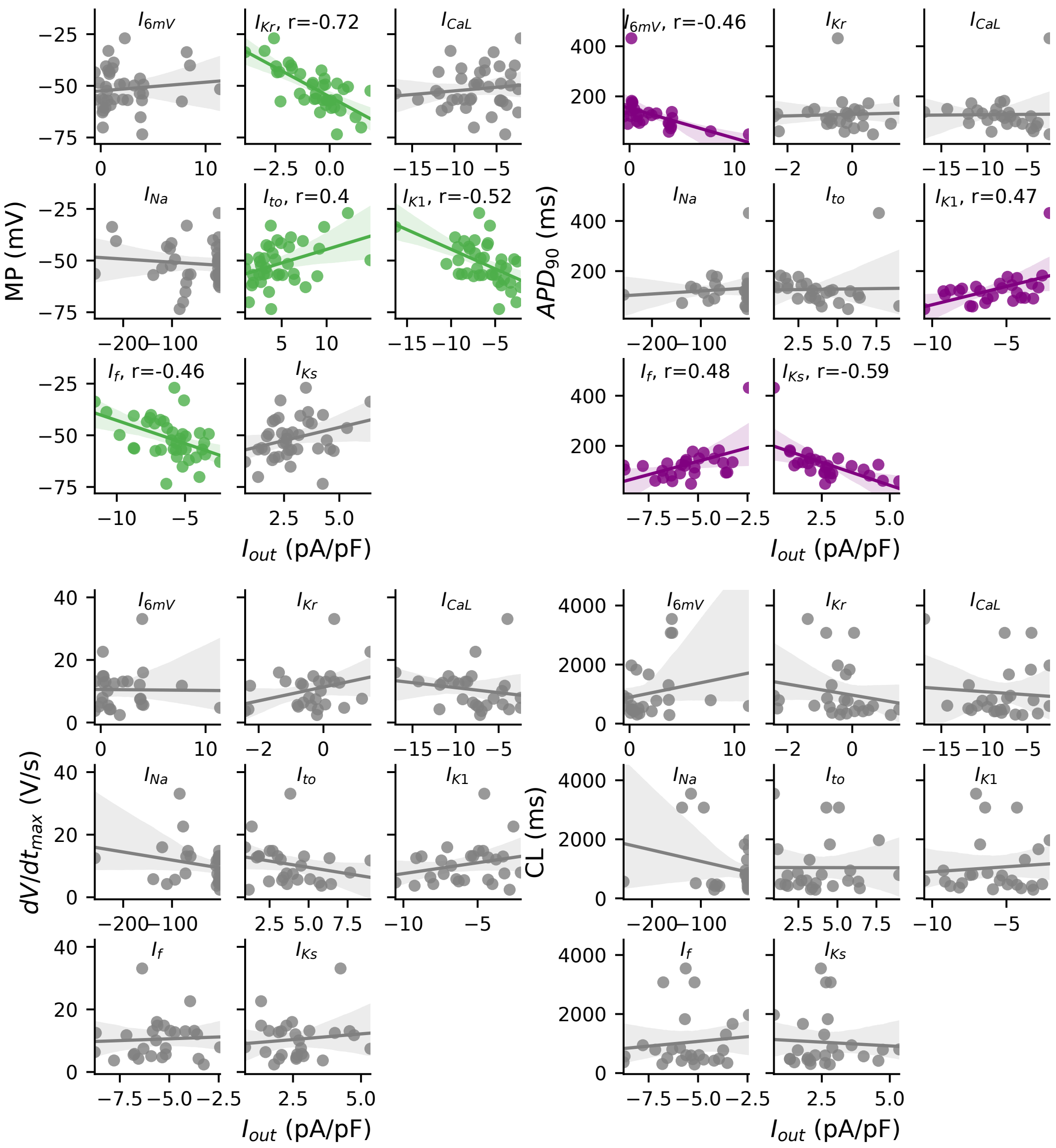

Figure 2: **AP parameter and current correlations for *in vitro* cells**. Correlations between AP metrics and current-isolating segments. Non-gray colors indicate correlations that met threshold for statistical significance.

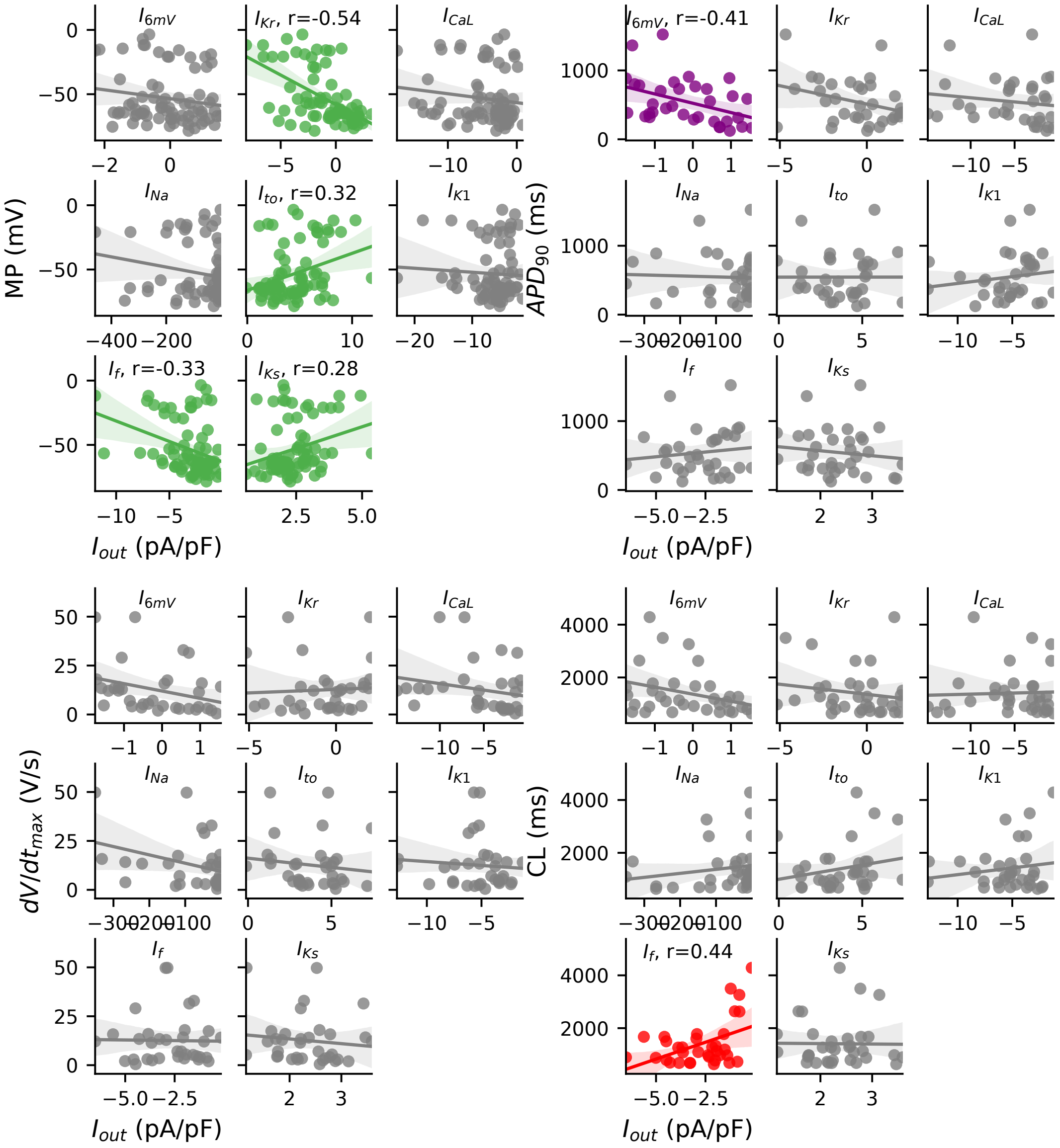

Figure 3: **AP parameter and current correlations for Paci-based model population**. Correlations between AP metrics and current-isolating segments. Non-gray colors indicate correlations that met threshold for statistical significance. There are no statistically significant correlations between dV/dt_{max} and any of the current-isolating segments. I_{Kr} is the main determinant of MDP, I_f the main determinant of CL, and I_{6mV} the main determinant of APD_{90}.

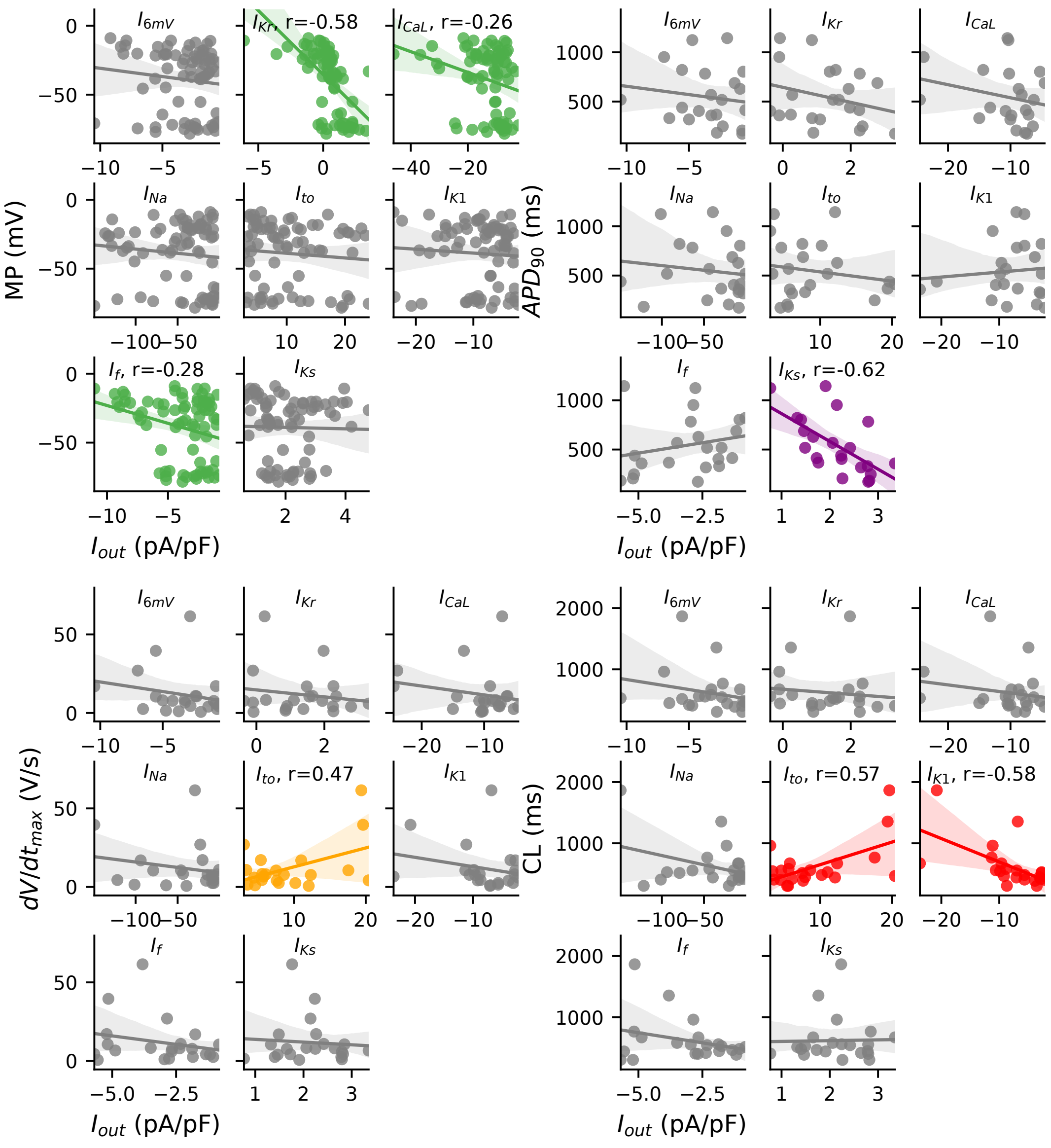

Figure 4: **AP parameter and current correlations for Kernik-based model population**. Correlations between AP metrics and current-isolating segments. Non-gray colors indicate correlations that met threshold for statistical significance. I_{to} correlate with dV/dt_{max}, I_{Ks} with APD_{90}, and both I_{to} and I_{K1} with CL. I_{Kr} is the main determinant of MDP.
